# Addressing Health Equity Through Action on the Social Determinants of Health: A Global Review of Policy Outcome Evaluation Methods

**DOI:** 10.15171/ijhpm.2018.04

**Published:** 2018-02-06

**Authors:** Janice Lee, Ashley Schram, Emily Riley, Patrick Harris, Fran Baum, Matt Fisher, Toby Freeman, Sharon Friel

**Affiliations:** ^1^School of Regulation and Global Governance (RegNet), College of Asia and the Pacific, Australian National University, Canberra, ACT, Australia.; ^2^Menzies Centre for Health Policy, Sydney Medical School, The University of Sydney, Sydney, NSW, Australia.; ^3^Southgate Institute of Health, Society and Equity, Flinders University, Adelaide, SA, Australia.

**Keywords:** Policy Evaluation, Methodology, Health Inequities, Social Determinants of Health, Review

## Abstract

**Background:** Epidemiological evidence on the social determinants of health inequity is well-advanced, but considerably less attention has been given to evaluating the impact of public policies addressing those social determinants. Methodological challenges to produce evidence on policy outcomes present a significant barrier to mobilising policy actions for health equities. This review aims to examine methodological approaches to policy evaluation of health equity outcomes and identify promising approaches for future research.

**Methods:** We conducted a systematic narrative review of literature critically evaluating policy impact on health equity, synthesizing information on the methodological approaches used. We searched and screened records from five electronic databases, using pre-defined protocols resulting in a total of 50 studies included for review. We coded the studies according to (1) type of policy analysed; (2) research design; (3) analytical techniques; (4) health outcomes; and (5) equity dimensions evaluated.

**Results:** We found a growing number of a wide range of policies being evaluated for health equity outcomes using a variety of research designs. The majority of studies employed an observational research design, most of which were cross-sectional, however, other approaches included experimental designs, simulation modelling, and meta-analysis. Regression techniques dominated the analytical approaches, although a number of novel techniques were used which may offer advantages over traditional regression analysis for the study of distributional impacts of policy. Few studies made intra-national or cross-national comparisons or collected primary data. Despite longstanding challenges of attribution in policy outcome evaluation, the majority of the studies attributed change in physical or mental health outcomes to the policy being evaluated.

**Conclusion:** Our review provides an overview of methodological approaches to health equity policy outcome evaluation, demonstrating what is most commonplace and opportunities from novel approaches. We found the number of studies evaluating the impacts of public policies on health equity are on the rise, but this area of policy evaluation still requires more attention given growing inequities.

## Background

While the epidemiology of the social determinants of health and health inequity is well-established,^1,2^ the body of research that evaluates the impact of multi-sectoral public policies on inequities in health risks and outcomes appears to be small and in its infancy.^3^ Historically, much of the policy evaluation literature has focused on formative and process evaluations with less emphasis on impact and outcome evaluations.^4,5^ The emphasis on formative evaluation has necessitated a reliance on qualitative techniques, including observations, interviews, and case studies, with occasional employment of quantitative techniques to compliment these methods.^6,7^ Policy outcome evaluation, on the other hand, requires the use of quantitative techniques, with inherent difficulties that are well documented. These include how to address complexity, attribution, selection effects, unobserved variables, time-lags, and data limitations in the research design when attempting to causally link policy to, often very distal, health outcomes.^8-11^

Notably, a lack of evidence on what works is commonly cited as a barrier to policy action on health inequities,^12^ even though such evaluations are important to inform future policy planning and implementation,^13^ and can support the efficient and effective use of limited public resources.^14^ Encouragingly there have been calls for outcome evaluation research from global and national level policy-makers. The World Health Organization (WHO) recommended to ‘measure and understand the problem and assess the impact of actions, specifically highlighting the importance of carrying out evaluations that measure how policies are effective in reducing social and health gradients.’^15^ It has also been asserted that quality policy outcome evaluation requires selection of a robust and well-suited methodology which should explore counterfactuals, quantify impacts across different levels of policy implementation and different population groups, study both direct and indirect effects, control for confounding factors and self-selection, and ideally be replicable by third parties.^16^

This article responds to these policy calls to undertake evaluation of public policy to highlight promising evaluation approaches, offer guidance on, and stimulate interest in, conducting future health equity policy evaluations, and identify the limitations of the existing methods used. We aimed to conduct a review of the literature to identify the range of methodological approaches being used to evaluate the health equity outcomes of public policy related to the social determinants of health. The focus of our review was on the methodological approach used within a study, rather than the substantive knowledge gained about the actual policy area being evaluated. In the review, we critically assessed the different approaches to study design, data collection methods, types of analyses, and the dimensions of health inequity evaluated. We defined health inequities as differences in health that are avoidable and unfair. However, we recognise that the term ‘health inequities/health equities’ is not universally used and hence are also interested in identifying methods used to evaluate health inequalities. This work is part of a broader research program, the ‘National Health and Medical Research Council Centre for Research Excellence on the Social Determinants of Health Equity’ (hereafter the CRE), which is examining a range of policies and policy processes (agenda setting, policy formulation, implementation and evaluation) and health equity.

## Methods

### Review Strategy

We undertook a narrative review of published materials following the guidance of Green, Johnson, and Adams on producing systematic narrative reviews for peer-reviewed journals, including conducting a preliminary scan of the literature to establish a need for the review; selecting appropriate databases; establishing search terms; defining inclusion and exclusion criteria; and designing an extraction table to guide our review and synthesis.^17^ We chose a narrative review because the flexibility allows for broader coverage of a wide range of issues within a given topic.^18^ At the same time, it summarises and interprets studies from which a contextual interpretation could be drawn from the reviewers’ own experience.^19^ The systematic search process ensures a rigorous approach to search for all possible and relevant research study. Five of the researchers participated in the review strategy: the search process (JL), study selection (JL, ER, SF, PH), and data extraction (JL and AS).

An initial search was first conducted from March to April 2016 to test the search terms, time period and the limits applied to electronic database. This search was conducted in Scopus and Web of Science to test the search combinations, and subsequently refined to enhance sensitivity to the target article content. The initial search terms were developed by one of the primary investigators (JL) and validated with the larger research team.

A conceptual framework was used to guide the selection of search terms. It is an adapted version of the ecosocial framework from the WHO Commission on the Social Determinants of Health.^20^ This was chosen because it has been widely used to understand the societal level factors that affect health inequities, and has contributed to the development of government policy and intersectoral action. The core elements of the framework include the structural drivers of power, money and resources and the conditions of daily living. The framework recognises that inequities in people’s daily living conditions are shaped by fiscal, labour, trade, social, land use and health policies; and cultural norms and values including gender norms and racism, which generate and distribute power, income, goods and services. Together, these structural factors and daily living conditions constitute the social determinants of health inequity.

We started the search with the term ‘social determinants of health’ and categories of social determinants of health (eg, marginalised, education, income, employment) alongside search terms specific to policy, equity, outcomes, and evaluation. Since the initial search terms were generating unmanageable search results (more than 10 000 papers were identified), we decided to use the term ‘social determinant’ to capture the broad aspect of social determinants of health. This refinement identified publications from a range of policy areas, particularly from social policy. Also, during the refinement process we added search terms specifically related to four policy domains based on the CRE focus noted previously (infrastructure [national broadband policy; land use and urban planning policy]; health systems [primary healthcare policy]; and Indigenous peoples policy). For the evaluation category, we started with the term evaluat* and expanded to others search terms such as measur*, assessment and quanti* based on the key words used in the articles during the refinement process (see Table 1 for the list of final search terms used).

**Table 1:** Summary of Search Strategy

**Database Searches:** additional limiting factors applied to each database during the final search	Proquest	Limit to search to Full-text and Peer reviewed Limit source types to Dissertations & Theses, Scholarly Journals, Government & Official Publications, reports
	Scopus	Limit document type to Article
	Web of Science	Limit search terms to Topic
	ScienceDirect	Limit to Journal search and Articles only Limit journals to Economics, Econometrics and Finance, Medicine and Dentistry, Nursing and Health Professions, Social Sciences
	PubMed	Search terms applied to all field
	Google Scholar	Mark Petticrew; Jennie Popay; Tony Blakely; David Ogilvie; Arjumand Siddiqi; David Stuckler; Margaret Whitehead; Alan Shiell; Paula Braveman; Barbara Starfield; John Lynch; Johan Mackenbach
**Search Terms**	Policy	Policy; Plan; Program*; Intervention; Service; System; Strategy
	Social determinants of health	Social Determinant; Urban; Urban Planning; Built Environmen; Racism; Aborig*; Indigen*; Primary Healthcare; Primary Health Care; Digital; Internet; Ehealth; E-Health; Telehealth; Broadband
	Equity	Equit*; Inequit*; Equalit*; Inequal*; Disparit*; Disadvant*
	Outcome	Health Outcomes; Health Impacts; Health Effects
	Evaluation	Evaluat*; Measur*; Quanti*; Assess*
	Evaluation types	Systems Modelling; Health Impact Assessment; Natural Experiments; Econometric; Multilevel Analysis; Structural Equation Modelling; Causation Modelling; Attribut* Analysis Or Contribut* Analysis

The time period used in the scoping review included papers published since 1990, however, the majority of the relevant papers appeared after 2000. For the actual search strategy, we limited the search to retrieve papers published since 2000 to the date of the search (29 April 2016). We repeated the search again on August 23, 2016 with more search terms on primary healthcare and broadband. We searched five multidisciplinary bibliographic databases: Web of Science, Scopus, ProQuest, PubMed, and ScienceDirect. An additional targeted search was undertaken at the end of the formal search strategy (12 October 2017) using the Google Scholar database for the work of known authors in the field as identified by the authors (see Table 1). All literature identified in the search were managed by bibliographic software (EndNote version X7) to eliminate duplicates and facilitate the reviewing process.

### Study Selection and Criterion

The primary screening of titles and abstracts of all identified papers for their eligibility was conducted by two authors (JL and ER). If it was unclear from the title and abstract whether the article met the above mentioned criteria, the article was included for a full-text review. A secondary screening of the full-text of eligible primary studies was completed by two authors (SF and PH), with discrepancies resolved in discussion with two other authors (JL and ER).

As the review focused on evaluations of public policy, an important step in developing the inclusion and exclusion criteria was to come to a shared understanding of the term public policy, which currently has no single definition.^21^ Public policy has been defined previously as the “…political decisions for implementing programs to achieve societal goals”^22,p1 ^, as “…the sum of government activities, whether acting directly or through agents, as it has an influence on the life of citizens”^23,p4 ^, and as “a statement by government of what it intends to do such as a law, regulation, ruling, decision, order, or a combination of these”^21,p8 ^.

Based on the abovementioned, we accepted public policy to include the declared objectives which a government seeks to achieve or a considered plan of action that a government intends to use to guide and determine its actions to achieve societal goals; implemented through a variety of policy instruments including legislation, regulation, rulings, papers and position statements, policies, plans, programmes, and projects.

From this it was decided a study would be included in the review if:

(1) it clearly indicated an evaluation was conducted of health equity impacts of the implementation of a public policy, or it clearly indicated that an evaluation was conducted of health equity impacts of a programme or project linked to implementation of a public policy, or consensus was reached by four team members (JL, SF, PH, and AS) that evaluation was conducted of health equity impacts of a programme or project which had the characteristics of being long-term and government-funded or initiated, and which appeared to be linked to the implementation of a public policy, though not explicitly stated. Any disagreements were resolved through discussion with all authors;

(2) it clearly indicated the methodology or types of method(s) used in the evaluation;

(3) it was peer-reviewed;

(4) it was in English;

(5) it needed to specify the health outcome measurement in the paper, and include direct or indirect relationship to health outcomes and health (in)equities.

A study was rejected if:

(1) it addressed the effectiveness or evolution of a public policy without measuring its impact were excluded;

(2) it did not include an empirical study (eg, was an opinion piece, commentary, letter, book review), review papers and conceptual and theoretical frameworks were excluded, but were checked for relevant references;

(3) it was not in English;

(4) the full-text was not available.

### Synthesis Strategy

Information such as author, year of publication, location of study, policy or policy instrument, study design, target population, dataset, outcome measurement, and analytical techniques were first extracted and entered into a matrix table of study characteristics. Data extraction was carried out by two authors (JL and AS) with uncertainties resolved by a third author (SF). Based on the data extraction, we then analysed studies to identify common characteristics between types of policy and methodological approaches used to measure health equity. Based on the commonality, we further developed an iterative coding schema over the course of the review and applied it for each of the studies (see Table 2). The codes included:

**Table 2:** Coding Scheme Used in Analysis

**Policy Area:** indicates the broad class of social or structural determinants of health inequity targeted by the policy under evaluation	*Environment/Living Conditions:* primary target of policy included living and working conditions, eg, smoke-free environments policies, healthy housing policies *Health System Management/Health Insurance:* primary target of policy included the management of or access to health systems, *eg,* national policies on subsidies to reduce user fees, national policies on contracting basic healthcare services *Social Protection and Welfare:* primary target of policy included national social services, eg*,* federally-funded retirement benefits policies, minimum income benefits policies *Taxation:* primary target of policy included taxation strategies, eg, tobacco taxation policies, policies regarding progressive taxation revenue sources
**Policy Target:** indicates the level at which the policy under evaluation was intended to operate	*Upstream:* policy targeted macro level factors (eg, income supplements) *Midstream:* policy targeted intermediate level factors (eg, health behaviours) *Mixed:* policy targeted both upstream and midstream level factors
**Study Design:** indicates the type of relationship explored between variables	***OBSERVATIONAL: seeks to establish association, researcher does not intervene or manipulate variables*** *Cross-Sectional*: data explored at one point in time *Longitudinal*: data explored at multiple points in time *Case-Control*: data explored on individuals with a specific condition (cases) and individuals without a specific condition (controls)
	***INTERVENTIONAL: seeks to establish cause-and-effect, researcher introduces or manipulates independent variable to observe change in a dependent variable*** *Experimental*: data explored on individuals who are randomly assigned to an experimental or control group and then compared on an outcome variable *Quasi-Experimental*: data explored on individuals in an intervention and a comparison group, without random assignment, and then compared on an outcome variable. *Pre-Experimental*: data explored on a group of participants after the introduction of an intervention or stimulus; however, lacks either baseline measurements or a control group for comparison *Natural Experiment*: data explored on individuals in an intervention and a comparison group, without random assignment, and then compared on an outcome variable, where the intervention is assigned by a policy or other exogenous socio-environmental change, not by the researcher
	***OTHER*** *Modelling*: process of producing a representation of the construction and working of a system of interest, similar to but simpler than the one it represents, for the purpose of predicting the effect of changes to the system *Simulation*: a tool to evaluate the performance of a system (modelled) under different configurations of interest and over long periods of real time. Microsimulation modelling examines individual units in the population and macrosimulation modelling examines proportions in the population *Meta-Analysis*: systematically combining pertinent qualitative and quantitative study data from several selected studies to develop a single conclusion that has greater statistical power
**Geographic Scope:** indicates the geographic range of data collection and comparison	*Localised* ***: ***data explored in one area of one country *Intra-National Comparison**: ***data explored in more than one area of one country *Cross-National Comparison**:*** data explored in more than one country
**Datasets:** indicates key details of the dataset(s) developed or utilised	***TYPE*** *Cross-sectional*: sample of the population at one point in time only *Repeated cross-sectional*: sample of a population at more than one point in time where n1 ≠ n2 *Cohort*: sample of a population at more than one point in time where n1 ≠ n2 but share a defining characteristic *Longitudinal*: sample of the same population at more than one point in time where n1 = n2 ***NUMBER *** *Single*: data analysed produced from a single data collection source *Multiple*: data analysed produced from multiple data collection sources ***TIMESPAN*** Earliest year of collected data used in analysis to latest year of collected data used in analysis, as combined across datasets (delineated when pre and post policy implementation) ***COLLECTION*** Primary: Data collected for purposes of policy evaluation Secondary: Externally collected data sourced for policy evaluation Mixed: Both primary and secondary data collection used for policy evaluation
**Analysis:** indicates the statistical techniques used to describe and illustrate, condense and recap, or evaluate the data	***Descriptive Analysis*** Graphical analysis (eg, boxplots, scatterplots, bar graphs, forest plot, etc); statistical summaries (eg, mean, standard deviation, median, interquartile range, odds ratio, etc); principal components analysis ***Inferential Analysis - General*** Comparison of means (eg, t test, ANOVA); chi-square; spatial autocorrelation ***Inferential Analysis - Regression*** Simple regression, multivariate regression, propensity score matching with regression, difference-in-difference regression, difference-in-difference-in-difference regression, fixed effects regression, instrumental variable with regression, regression discontinuity, survival analysis, meta-regression, change-on-change, generalised estimating equations regression, decomposition regression; hierarchical lineal regression ***Decision Analysis*** Stochastic Dominance ***Mixed Methods Analysis*** Qualitative comparative analysis (eg, crisp-set, fuzzy-set) ***Modelling*** Microsimulation modelling; macrosimulation modelling; multistage lifetable modelling ***Meta-Analysis*** Harvest plot
**Outcome**: indicates the outcomes analysed	*Short-term outcome*: measure of attitudes, knowledge, or beliefs *Intermediate outcomes*: measure of health behaviour or accessibility, availability, or affordability of health-related products or services *Long-term outcome*: measure of physical and mental health outcome, including self-report, metabolic or physiologic indicators, and morbidity and mortality rates
**Inequity Dimension:** indicates which dimension of inequity was targeted by the policy or which dimension of inequity was explored in the policy evaluation	*Main dimensions of inequity (plus frequently employed subcategories where applicable):* 1. Age a. youth b. seniors 2. Education 3. Health a. poor health b. availability of health services 4. Income a. deprivation b. LMICs c. wealth 5. Occupation a. unemployed b. precarious employment c. low-payed employment 6. Place of Residence a. disadvantaged area b. rural/urban 7. Race/Ethnicity a. Indigenous 8. Sex a. single-mothers 9. Sexual Orientation 10. Socio-Economic Status

(1) policy - the broad type of policy area targeted by the policy under evaluation (eg, social protection and welfare, taxation) and whether the policy was targeted at upstream level (eg, income supplements), or midstream level (eg, health behaviours), or was a mix of the two;

(2) methodological approach – the type of study design, the geographic scope, data collection, analysis, outcomes, and inequity dimensions measured.

The definition used to identify the policy target (upstream, midstream or a mix) was in accordance with Carson et al.^24^ For the coding of outcome measurement, we classified it according to short-term, intermediate, and long-term outcomes as listed in the framework to monitor and evaluate implementation by the WHO.^25^ For the classification of equity dimension, we did not refer to any reference but relied on the common inequity dimensions measured by the studies. We created subcategories of inequity dimensions if the study mentioned a specific dimension of measure. For example, when studies were stratified according to specific ethnic groups, we would code them as Race/Ethnicity. However, when the studies were stratified as indigenous and non-indigenous, we would code it as Race/Ethnicity – Indigenous.

## Results

**Figure 1:**
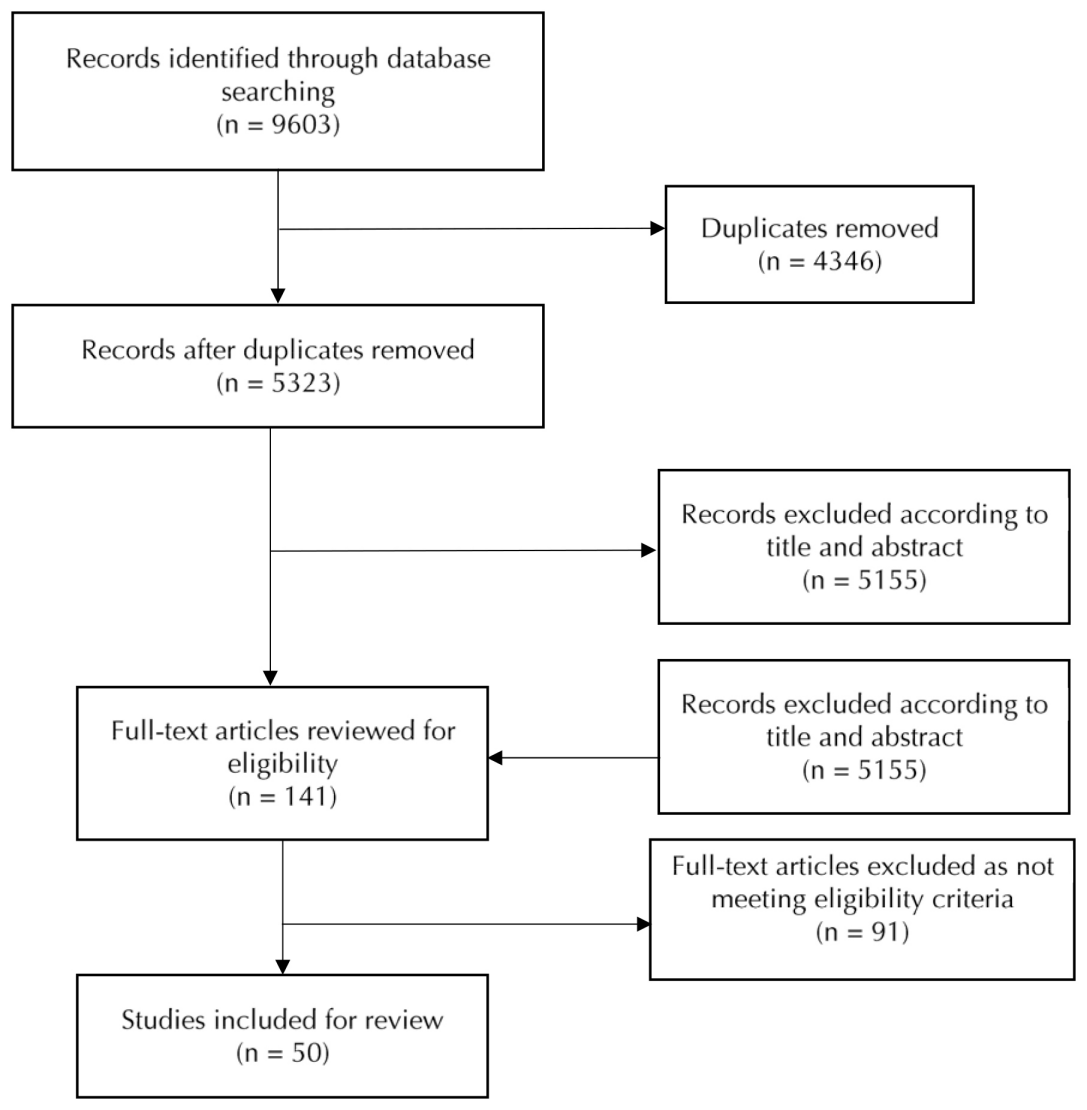
Flow Chart of the Review Selection Process.

The electronic database identified 5289 publications of which 135 were deemed as potentially eligible. After screening, 50 papers were eligible for inclusion in this review (see Figure 1). A summary and the results of the coding of the included studies are present in Supplementary file 1. The dates of the publications ranged from 2002 to 2016.

### Research Designs Used in Evaluations

Of the 50 outcome evaluation articles reviewed, just over half employed an observational research design (n = 26, 52%), most of which were cross-sectional (n = 15, 30%). The remaining studies reviewed used more innovative research designs. These included establishing the outcome evaluation as a form of experimental design and viewing the policy as an intervention (n = 19, 38%); using a form of simulation modelling to model the predicted impacts of one or more policy interventions on a population (n = 4, 8%), and the use of a meta-analytical design to synthesise evidence across multiple intervention evaluations (n = 1, 2%) (see Table 3).

**Table 3:** Frequency of Articles by Policy and Research Design

**Policy**	Policy area		Social orotection and welfare	17	34%
			Health system management/ health insurance	16	32%
			Environment/living conditions	14	28%
			Taxation	3	6%
	Policy target		Upstream	41	82%
			Midstream	3	6%
			Mixed	6	12%
**Research Design**	Study design	Observational	Cross-sectional	15	30%
			Case-control	2	4%
			Longitudinal	9	18%
		Interventional	Pre-experimental	10	20%
			Quasi-experimental	1	2%
			Natural experiment	7	14%
			Experimental	1	2%
		Other	Modelling and simulation	4	8%
			Meta-analysis	1	2%
	Geographic scope		Localised	31	62%
			Intra-national comparison	8	16%
			Cross-national comparison	10	20%
			Not Specified	1	2%
	Number of datasets		Single	22	44%
			Multiple	28	56%
	Type of datasets		Cross-sectional	6	12%
			Repeated Cross-sectional	29	58%
			Cohort	4	8%
			Longitudinal	10	20%
			Not specified	1	2%
	Data collection		Primary Secondary Mixed	5 44 1	10% 88% 2%

### Policy Area and Target of Evaluations

The policy areas targeted by evaluations were: social protection and welfare policies (n = 17, 34%), health systems management and health insurance policies (n = 16, 32%), environment and living conditions policies (n = 14, 28%) (see Table 3) and taxation policies (n = 3, 6%). Moreover, two of the three evaluations of taxation employed simulation modelling^26,27^; suggesting perhaps evaluations in this policy area may be more suitable for advanced quantitative analyses, although this inference should be interpreted with caution given the small sample. Almost all of the reviewed polices were designed to act on macro or structural level factors (n = 41, 82%). This is likely an artefact of our search terms and inclusion criteria, as many evaluations of interventions at the individual behavioural level may have been presented as program evaluations without any clear connections to a specific public policy within the publication.

### Geographic Scope of Evaluations

**Figure 2:**
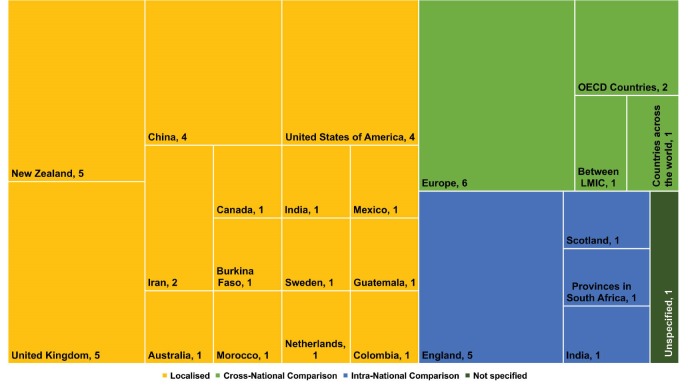
A TreeMap of the Studies by Geographical Scope.

Most of the studies evaluated outcomes in one geographical area (localised, n = 31, 62%), although a number did make comparisons across areas in a single country (intra-national, n = 8, 16%) or across multiple countries (cross-national, n = 10, 20%) (see Table 3 and Figure 2).

**Figure 3:**
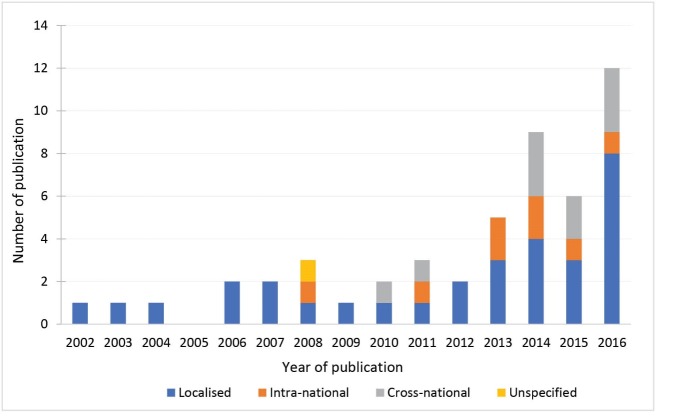
A Chart of the Frequency of Publications and the Studies’ Geographical Scope by Year.

An interaction emerged between the geographic scope selected for the evaluation, the year of publication, and the type of policy area targeted (see Figure 3). The number of studies increased rapidly from 2013 onwards. Most of the intra-national and cross-national comparison evaluation studies were conducted in the last four years. Specifically, eight of the 10 cross-national comparisons explored social protection and welfare policies. Five of the eight studies using intra-national comparisons examined environment and living condition policies. Cross-national comparisons in this policy area may present considerable challenges to the internal validity of the study (given the likelihood of there being a higher degree of variability in environmental and living conditions across countries relative to within countries), but where variations occurred within a country this may be of interest to evaluators, particularly in exploring area-related health inequities. While a localised study design was utilised in all policy areas, the use of this design was almost ubiquitous in the area of health system management or health insurance policies, with fourteen of the sixteen studies localised to either one country or one area within a country. The two exceptions to this were both intra-national comparisons – one in England exploring how changes in the National Health Service (NHS) affected geographical areas of varying affluence^28^; and one in South Africa that compared disease burden in varying race and income groups by provincial public health funding allocations.^29^

### Datasets Used in Evaluation

One of the challenges of policy outcome evaluation is accessing suitable data. The evaluations we reviewed were split almost evenly on whether they were able to find a single data source suited to the purpose (n = 22, 44%) or were required to weave together multiple data sources (n = 28, 56%) (see Table 3). Cross-sectional and repeated cross-sectional datasets were dominant (n = 35, 70%), with 10 studies (20%) employing a longitudinal dataset (see Table 3). six studies (12%) in our review collected primary data (one in conjunction with additional secondary data), suggesting that primary data collection is rare in policy outcome evaluation (see Table 3). Five of six studies that collected primary data were localised geographically, indicating such data collection is less feasible for intra-national and cross-national comparisons. Additionally, four of those same six studies involved evaluation of environment and living condition policies targeting individual behavioural change, such as a community intervention to improve health-related behaviour among adults living in deprived neighbourhoods through activities largely directed at nutrition or physical activity.^30^ Public policy implemented at a community level targeting individual behavioural change may be more likely to have an evaluation component and linked data collection if the relationship between implementation activities and health equity outcomes are perceived as more proximal.

### Outcomes Used in Evaluation

Given the oft-discussed challenges of making attributes in policy outcome evaluation, it was interesting to find that 36 studies (72%) included a measure of either physical or mental health. Examples included evaluating the impact of welfare state arrangements on self-rated health among single-mothers and the unemployed^31^; anti-bullying policies on self-reported suicide attempts in homosexual and bisexual youth^32^; or gender equity in labour policies on self-perceived health.^33^ Moreover, physical or mental health outcomes were more commonly used than either health-risk factors (n = 29, 58%, ie, a measure of health behaviour or accessibility, availability, or affordability of health-related products or services) or personal factors (n = 4, 8%, ie, a measure of attitudes, knowledge, or beliefs).

The continued need for evidence-based policy-making has fuelled theory-based evaluation and program logic approaches to mitigate some of the challenges of outcome evaluation, such as attribution or intervening variables.^9,10,34,35^ Examples included theory of change approaches, which explore how and why initiatives do or do not work^36^; and realist evaluation, which begins with a theory of change and uses multiple methods to ascertain what it was about the wider contextual influences and the mechanisms of the intervention that resulted in improved outcomes or not.^37^ While four studies in our review included a figure detailing a theory of change or policy logic model guiding the evaluation^31,38-40^; explicit incorporation of a theory-based evaluation approach to address the challenge of attribution was minimal in the studies we reviewed.

### Equity Dimensions Included in Evaluation

Place of residence was the most frequent equity dimension measured (n = 22, 44%), followed by sex and income (n = 16, 32% for both), occupation (n = 14, 28%), age (n = 12, 24%), education, race and ethnicity (n = 11, 22% for both), and sexual orientation (n = 1, 2%). A small selection of studies (n = 4, 8%) utilised pre-existing health status as a dimension of equity (n = 4, 8%) based on the rationale that differences in health status can affect the social determinants of health, such as employment status.^41^

### Analysis Used in Evaluation

**Figure 4:**
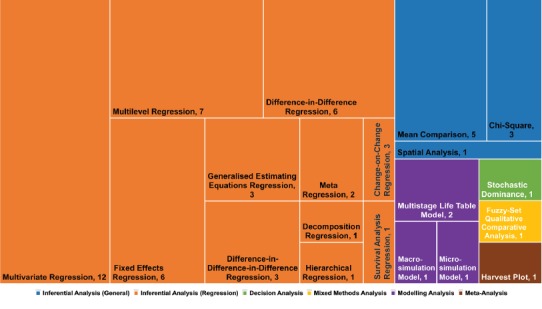
TreeMap of Analytic Techniques.

A key area of interest to us was the type of analysis used to undertake the evaluation. While all studies employed descriptive analysis (eg, means, standard deviations) and over half (n = 33, 66%) included a form of graphical analysis (eg, scatterplots, forest plots), five of the studies (10%) employed only descriptive analyses to evaluate the outcomes of policy implementation. General inferential analysis, including mean comparisons and chi-square, were infrequently used (n = 9, 18%) (see Figure 4). One novel technique in this category included a spatial autocorrelation analysis of geographic information system data to assess the spatial equity of amenities, resources, and infrastructure dedicated to obesity interventions.^42^

The dominance of regression techniques in outcome evaluation was apparent, a foreseeable finding given its central role in the quantitative public health analysis landscape. Regression techniques were used 43 times in 35 (70%) different studies. The most frequent types of regression models used included a multivariate regression model (n = 12, 24%), a multilevel regression model (n = 7, 14%), a difference-in-difference regression model (n = 6, 12%), or a fixed effects regression model (n = 6, 12%) (see Figure 2). There were no clear patterns in which types of research designs or policy areas were likely to use or not use regression techniques. Most of the regression analyses employed techniques to strengthen their ability to suggest causal claims, such as the use of multiple control variables, inclusion of contextual level factors, well-constructed control groups or use of synthetic controls, as well as a number robustness checks to assess model fit.

A small number of analytical approaches fell outside traditional inferential statistics including those using simulation modelling (n = 4, 8%); decision analysis with stochastic determination (n = 1, 2%); mixed methods analysis with fuzzy-set qualitative comparative analysis (n = 1, 2%); and a newly-developed meta-analysis technique termed a harvest plot (n = 1, 2%). The use of simulation modelling, such as that performed by Basu and colleagues^26^ to model the projected impact of a 20% sugar-sweetened beverage tax on increases in overweight, obesity, and type 2 diabetes cases, was a pertinent technique for projecting the impacts of suggested policy changes. The study from Van de Gaer and colleagues used stochastic dominance, a form of decision analysis, to evaluate the effectiveness of a conditional cash transfer program on children’s health based on parental education level and indigenous status – rather than examining average treatment effects as is often the case.^43^ McNamara used fuzzy-set qualitative comparative analysis, a method which identifies populations with “successful” outcomes and explores combinations of policy implementation factors to understand what parts of interventions work under which contexts^40^; this is a particularly useful analytical tool for realist evaluation approaches that develop similar explanatory propositions.^37^ Finally, Ogilvie et al demonstrated a methodological innovation in this area by developing the harvest plot – an adaptation of the forest plot to account for evidence from a complex and diverse group of studies investigating outcomes along various dimensions of inequality.^44^ Sophisticated regression modelling and novel analytical techniques, such as those noted above, provide new ways forward to overcome the noted challenges of policy outcome evaluation.

## Discussion

The results of our review demonstrate the growing body of work evaluating the impact on inequities in health outcomes of public policy that addresses the social determinants of health. We identified the types of policies undergoing evaluation and the variety of research designs employed to meet the unique needs of evaluating complex policy on health equity outcomes. The studies in our review show considerable use of experimental research design in policy evaluation, underscoring the importance of further development of such.^10^ We note that some methodological approaches, such as designing geographical comparisons, may be more or less suitable depending on the policy areas undergoing evaluation, suggesting a one-size fits all approach to policy evaluation is likely to be inappropriate.

There is also an expansion in evaluation studies to broaden the scope of evaluation studies between different countries or areas within a country since they were conducted more recently. A reason for this could be the availability of data sources or policies that are comparative. Most of the cross-national or intra-comparison studies were focused in countries within Europe, making it a possibility that these countries have similar data sources. For example, there is a common data source in Europe, called the European Social Survey, which is an academically driven biennial cross-national survey.^45^ This survey was designed to collect cross-national data on social and political attitudes, conditions as well as outcomes in Europe. The reliability, quality and availability of this data source, and the representativeness of the data sample makes this regional multi-purpose survey appropriate for policy evaluation.^46,47^

Another reason could be that the policy areas, such as social or welfare policies, were more comparative as compared to other regions which have marked differences in the same policy areas. This could also imply why eight out of ten of the cross-national comparison studies were focused on the policy areas of social protection and welfare policies. International comparisons may be more viable with this particular policy area, as social protection and welfare policies are usually made at the national level (rather than provincially for example), and pre-existing welfare state typologies and international databases can be utilised to evaluate the impact of different types of social protection and welfare policies across countries on health equity outcomes. For example, almost all studies evaluating health system management or health insurance policies did not include any geographical comparisons and this may speak in part to the complexity of health systems and may suggest that this policy area is perceived to preclude valid international comparative evaluations.

Data collection across almost all studies was heavily reliant on secondary data sources. Employing secondary data can be entirely adequate in some evaluations; however, it inevitably introduces the possibility of selecting data based on what is currently available rather than what would be ideal for the evaluation. As macro or structural level policy change is rarely associated with any specific data collection efforts about health equity effects, this remains a challenge in outcome evaluation, and stresses the need for high-quality, linked, nationally-representative and routinely collected longitudinal databases. The reality, nevertheless, is that the quality and quantity of data currently collected is insufficient to execute these advanced analyses for many complex policy outcome evaluations.

The review also demonstrates that nearly three out of every four studies reviewed endeavoured to attribute changes in a measure of either physical or mental health to the design of, or change in, public policy. White notes that while some evaluators have suggested that attribution is rarely possible, for many others “…attribution is the defining characteristic [of an impact evaluation] especially those using quantitative methods to measure impact.”^48,p154^ Some authors have proposed quantitative methods to address the attribution problem. For instance, Leeuw and Vaessen describe techniques such as propensity score matching, difference-in-difference, regression analysis, instrumental variables, and regression discontinuity analysis as ways to overcome the attribution problem.^9^ In another example, White suggests the development of counterfactuals and investigating the underlying causal chain from inputs through to outcomes and impacts using a mixed methods approach.^48^ A number of documents have been developed to provide guidance on conducting impact evaluations of complex interventions^9,10,49^; on evaluating the impact of natural policy experiments on health and health inequities^50,51^; and on conducting evaluation within specific public policy areas.^52^

In addition to more robust quantitative analytical techniques, others advocate for theory-based realist evaluation approaches to understand how and why policies work, or do not work, for which populations, and in what contexts^35,37,52^ Studies in our review reflected these approaches for addressing the attribution problem, including many of the regression techniques suggested by Leeuw and Vaessen,^9^ and Hu and colleagues,^50^ as well as some engagement with theory-based evaluation. Our review shows that an integration of the various facets of outcome evaluation described above is required.

Analytical approaches that fell outside of traditional inferential statistics were also very recent studies, published within the last three years. The rise towards using more complex modelling and simulation techniques has the capacity to make substantial contributions to evidence-informed policy decision-making.^26^ We also found a study that applied stochastic dominance to evaluate equity impact and this type of analysis is promising for assessing the effects of policy on equity, in that it investigates equality of opportunity and focuses on the distributions that are conditional on circumstances, such as race or education, instead of comparing the distributions of all treatment and control samples.^43^ The limited number of studies that use innovative or more advanced analytical techniques could imply that in reality, the quality and quantity of data currently collected could be insufficient to execute these advanced analyses for many complex policy outcome evaluations. However, applying these techniques will facilitate evaluations of outcomes identified by international and national agencies and government as a priority for research.

We found a small number of studies evaluating taxation policies, even though it is an area where we might expect to see more outcome evaluations given its capacity for quantification. It could be a possibility that the very small number of studies found was a result of not including a term specific for taxation in our search strategy, hence we did not capture the full extent of evaluations in this area.

## Conclusion

Public policies are inherently complex, variable in practice, and their implementation can affect their impact on population health.^53^ The design of the policy evaluation is dependent on numerous factors such as availability and quality data, the representativeness of the sample size, the policy area, and the conditions of the policies (such as timing of the policies for before and after study or variance in timing to allow for natural experiments). However, it is beyond the scope of this review to determine which methodology is best suited or most appropriate to quantify impacts of policies on health equity. This is due to the way in which the mechanism as part of the effects of such policies would lead to an impact on health equity.^53^ However, we have shed light on what types of methodologies have been used to quantify the effect of policies on health equity.

A lack of data on health equity outcomes and evidence about what works to reduce them has been cited as a major obstacle to policy change.^12,54^ This paper aimed to contribute to the development of a body of evaluation addressing exactly that. The number of studies evaluating public policies’ impact on health equity are rising with more studies comparing policies within or across countries. Despite the growth, the number of studies we found is still relatively small and warrants more study. Accordingly, this paper and future work within the CRE is pursued on the premise that conducting the type of high-quality comprehensive outcome evaluations described above will continue to be essential to contribute to evidence-informed policy supported by larger socio-political transformations.

## Acknowledgements

This work was supported by the NHMRC Centre of Research Excellence on the Social Determinants of Health Equity: Policy research on the social determinants of health equity (APP1078046). The NHMRC had no role in the conduct of this research.

## Ethical issues

Not applicable.

## Competing interests

Authors declare that they have no competing interests.

## Authors’ contributions

All authors conceived the idea for the review through group discussion. JL undertook the systematic search. JL and ER screened the title and abstract, while SR and PH screened the full-text. JL and AS coded the final included studies, analysed the data, and wrote the first draft of the manuscript. All authors provided input into ongoing iterations of the manuscript and approved the final version.

## Authors’ affiliations

^1^School of Regulation and Global Governance (RegNet), College of Asia and the Pacific, Australian National University, Canberra, ACT, Australia. ^2^Menzies Centre for Health Policy, Sydney Medical School, The University of Sydney, Sydney, NSW, Australia. ^3^Southgate Institute of Health, Society and Equity, Flinders University, Adelaide, SA, Australia.

## Supplementary files

Supplementary file 1 contains Table S1.
