# Supplementary material for: Addressing Health Equity Through Action on the Social Determinants of Health: A Global Review of Policy Outcome Evaluation Methods
Source: Int J Health Policy Manag. 2018 Feb 6;7(7):581–92. doi: 10.15171/ijhpm.2018.04 (PMC6037500; doi:10.15171/ijhpm.2018.04)
Supplement: Supplementary file 1 — contains Table S1. [file ijhpm-7-581-s001.pdf]

Appendix 1

Table S1. Coding Results

**OBSERVATIONAL – CROSS SECTIONAL**		
Boutayeb et al. 2016<br><br>*Actions on social determinants and interventions in primary health to improve mother and child health and health equity in Morocco*	**Policy**	Actions and strategies in enhancing access to healthcare (in accordance to Millennium Development Goals)
	**Policy Area**	Health System Management/Health Insurance
	**Policy Target**	Upstream
	**Geographic Scope**	Localised (Morocco)
	**Dataset(s)**	Cross-sectional; Multiple datasets; 1992-2012; Secondary data
	**Analysis**	Descriptive analysis: graphical analysis + statistical summaries + principal components analysis; Inferential analysis: chi-square analysis
	**Outcome(s)**	<u>Intermediate</u>: access to drinking water, electricity and sanitation; healthcare expenditure per capita; number of basic healthcare facilities, public hospital, physicians, nurses and midwives; national health insurance coverage; percentage of skilled deliveries, prenatal visits, contraception and vaccination prevalence; vaccination; antennal care<br><u>Long term</u>: neonatal, infant and under-5, maternal mortality; proportion of stunting and malnutrition; children with diarrhoea and pneumonia; pregnant women with anaemia
	**Inequity Dimension**	Education + Income - wealth quintiles + Place of residence - urban/rural
	**Summary**	(1) Study compared the access to drinking water, electricity and sanitation; healthcare expenditure per capita; number of basic healthcare facilities, public hospital, physicians, nurses and midwives; national health insurance coverage; percentage of skilled deliveries, prenatal visits, contraception and vaccination prevalence; neonatal, infant and under-5, maternal mortality rates; proportion of stunting and malnutrition in children; pregnant women with anaemia before and after the Millennium Development Goals (following the evolution of healthcare system); comparing between rural/urban areas, mother's education level, wealth quintiles, and public and private section (2) Study measured the spatial disparity by comparing the percentage of antennal care, delivery assistance, contraception, vaccination, employed women, GDP per capita, children with diarrheal and suspected pneumonia, stunting children, young illiteracy, need in medical staff; under-5 mortality rate a ross 13 regions.
Burstrom et al. 2010<br><br>*Health inequalities between lone and couple mothers and policy under different welfare regimes – The example of Italy, Sweden and Britain*	**Policy**	Different welfare state arrangements and social policy in 3 contrasting family policy environment
	**Policy Area**	Social Protection and Welfare
	**Policy Target**	Upstream
	**Geographic Scope**	Cross-National Comparison (N=3)
	**Dataset(s)**	Repeated cross-sectional; Multiple data; 1999-2001; Secondary data
	**Analysis**	Descriptive analysis: statistical summaries
	**Outcome(s)**	<u>Long term</u>: self-rated general health, limiting longstanding illness

	**Inequity Dimension**	Occupation - unemployed + Sex - single-mothers
	**Summary**	Study compared the age-standardised prevalence rate of the self-rated general health and limiting longstanding illness of single and coupled women between the 3 different countries with contrasting family policy models (dual-earner/market oriented/general), considering the different countries social, cultural and traditions, stratified by occupation class and employment status.
Cristia, Prado and Peluffo 2015<br><br>*The impact of contracting in and contracting out basic health services: the Guatemalan experience*	**Policy**	Contracting and coverage extension of basic health care services (child and maternal)
	**Policy Area**	Health System Management/Health Insurance
	**Policy Target**	Upstream
	**Geographic Scope**	Localised (Guatemalan)
	**Dataset(s)**	Repeated cross-sectional; Single dataset; 1995, 2002; Secondary data
	**Analysis**	Descriptive analysis: graphical analysis + statistical summaries; Inferential analysis: difference-in-difference regression model
	**Outcome(s)**	Intermediate: presence of child immunization card; BCG, Polio, DPT, tetanus vaccination coverage; access to prenatal care; first trimester check-ups; use of family planning methods
	**Inequity Dimension**	Income + Place of residence - rural/urban + Race/Ethnicity - Indigenous
	**Summary**	Study measured the associations between contracting in and out the basic health service and child immunization (presence of child immunization card; BCG, Polio, DPT dose coverage); prenatal care and family planning (frequency of prenatal care visits; prenatal visit during first trimester; location of prenatal care service; tetanus vaccination; use of family planning), comparing between contracting in and contracting out; contracting in and not covered; contracting out and not covered.
Dalton et al. 2013<br><br>*Using spatial equity analysis in the process evaluation of environmental interventions to tackle obesity: the healthy towns programme in England*	**Policy**	Healthy towns programme (focus on interventions to provide dietary and physical activity behaviour change)
	**Policy Area**	Environment/Living Conditions
	**Policy Target**	Midstream
	**Geographic Scope**	Intra-National Comparison: England
	**Dataset(s)**	Cross-sectional; Single data; 2001; Secondary data
	**Analysis**	Descriptive analysis: graphical analysis + statistical summaries; Inferential analysis: spatial autocorrelation + analysis of variance
	**Outcome(s)**	Intermediate: spatial access to physical activity, food systems, healthy lifestyle, physical activity
	**Inequity Dimension**	Place of residence - disadvantaged area + Race/Ethnicity + Socioeconomic status
	**Summary**	Study measured the association between the spatial equity geographical distribution of each interventions and the targeting population in need by 1) calculating the targeting ratio to measure the ratio of good access of infrastructural provision of the programme to population in need against rest of the population with good access; (2) computing the

		geographical distribution of each population in need to measure geographical diversity of disadvantage populations; (3) measuring the equity of infrastructure provision using mean targeting ratios comparing the tertiles of clustering of the disadvantaged population by black and minority ethnic groups, children, socio-economic disadvantage and retired household.
Emery, Fleisch & McIntyre 2013<br><br>*Legislated changes to federal pension income in Canada will adversely affect low income seniors' health*	**Policy**	Federally-funded retirement benefits (raising age of eligibility from 65 to 67)
	**Policy Area**	Social Protection and Welfare
	**Policy Target**	Upstream
	**Geographic Scope**	Localised (Canada)
	**Dataset(s)**	Cross-sectional; Single dataset; 2007/2008; Secondary data
	**Analysis**	Descriptive analysis: graphical analysis + statistical summaries
	**Outcome(s)**	Intermediate: food insecurity
	**Inequity Dimension**	Age - seniors + Income
	**Summary**	Study compared proportion of unattached seniors with a personal income of less than $20,000 between the ages of 60-64 and 65-69 that are food insecure stratified by main source of income to forecast how the policy change may affect food insecurity in affected populations as an indicator of economic security and physical/mental health
Hatzenbuehler & Keyes 2013<br><br>*Inclusive Anti-bullying Policies and Reduced Risk of Suicide Attempts in Lesbian and Gay Youth*	**Policy**	Anti-bullying policies (inclusive of sexual orientation)
	**Policy Area**	Social Protection and Welfare
	**Policy Target**	Upstream
	**Geographic Scope**	Localised (Oregon, USA)
	**Dataset(s)**	Repeated cross-sectional; Single dataset (annual); 2006-2008; Secondary data
	**Analysis**	Descriptive analysis: statistical summaries; Inferential analysis: generalised estimating equations regression model
	**Outcome(s)**	Long-term: self-reported suicide attempts
	**Inequity Dimension**	Age - youth + Sexual orientation
	**Summary**	Study measured the association between anti-bullying policies (inclusive and non-inclusive of sexual orientation) and suicide attempts among lesbian and gay youths, bisexual youths, and heterosexual youths
Holland et al. 2011<br><br>*How do macro-level contexts and policies affect the employment chances of chronically ill*	**Policy**	Flexible and deregulation, decommodification and investment in labour market policies on employment of chronically ill and disabled people
	**Policy Area**	Social Protection and Welfare
	**Policy Target**	Upstream
	**Geographic Scope**	Cross-National Comparison (N=5)

and disabled people? Part II: The impact of active and passive labor market policies	**Dataset(s)**	Repeated cross-sectional; Multiple dataset; 2002-2005; Secondary data
	**Analysis**	Descriptive analysis: graphical analysis + statistical summaries; Inferential analysis: multivariate regression model
	**Outcome(s)**	Intermediate: age-standardised employment rates
	**Inequity Dimension**	Education + Health - poor health + Sex
	**Summary**	1) Study measured the associations between employment rates and various degree of flexible, deregulated labour market; different relative generosity and entitlement of welfare benefits (decommodification); and greater investment and emphasis on active labour market policies by comparing the age-standardised employment rates between healthy population with those with longstanding illness that was not limiting; or those with limiting longstanding illness, stratified by sex, education level and countries
Hu et al. 2016<br><br>*The impact of tobacco control policies on smoking among socioeconomic groups in nine European countries, 1990-2007*	**Policy**	Different tobacco control policies
	**Policy Area**	Environment/Living Conditions
	**Policy Target**	Midstream
	**Geographic Scope**	Cross-National Comparison (N=9)
	**Dataset(s)**	Repeated cross-sectional; Multiple dataset; 1990-2007; Secondary data
	**Analysis**	Descriptive analysis: graphical analysis + statistical summaries; Inferential analysis: fixed effects regression model
	**Outcome(s)**	Intermediate: Smoking prevalence
	**Inequity Dimension**	Education + Occupation + Sex
	**Summary**	1) Study compared the country trends in the price and non-price measures over time 2) Study compared the trends in age-standardised smoking prevalence by education in each country for men and women over time 3) Study measured the association between price (popular cigarette and cheapest cigarette price) and non-price related tobacco control policies, and smoking, comparing the odds ratio between man and women stratified by education level and occupation
McNamara 2015<br><br>*Trade liberalization, social policies and health: An empirical case study*	**Policy**	Social protective policies of welfare states and labour markets during trade liberalization (focus on employment changes of textile and clothing sector)
	**Policy Area**	Social Protection and Welfare
	**Policy Target**	Upstream
	**Geographic Scope**	Cross-National Comparison (N=32)
	**Dataset(s)**	Repeated cross-sectional; Multiple dataset; 2000-2009; Secondary data
	**Analysis**	Descriptive analysis: statistical summaries; Fuzzy-set qualitative comparative analysis
	**Outcome(s)**	Long term: Adult female mortality rates; infant mortality rates
	**Inequity Dimension**	Occupation - unemployed + Sex

	**Summary**	Study measured associations between countries' level of development, labour market and welfare state protections, and the sector's employment changes on mortality rates. Each countries are assigned membership score of health improving or health worsening based on 5-year period preceding and following the phase out period of the trade liberalization. Study used process-tracing analysis to further investigate these associations through 12 in-depth country studies.
Mosquera et al. 2012<br><br>*The impact of primary healthcare in reducing inequalities in child health outcomes, Bogotá - Colombia: An ecological analysis*	**Policy**	Comprehensive primary health care (home health program)
	**Policy Area**	Health System Management/Health Insurance
	**Policy Target**	Upstream
	**Geographic Scope**	Localised (Bogotá, Colombia)
	**Dataset(s)**	Repeated cross-sectional; Multiple datasets; 2003, 2007; Secondary data
	**Analysis**	Descriptive analysis: graphical analysis + statistical summaries; Inferential analysis: decomposition regression model
	**Outcome(s)**	<u>Intermediate</u>: diphtheria, pertussis, tetanus vaccination coverage<br><u>Long term</u>: infant mortality rate; under-5 mortality rate; prevalence of acute malnutrition in children under 5 years of age
	**Inequity Dimension**	Place of residence - disadvantaged area
	**Summary**	(1) Study measured the socioeconomic inequality using the outcomes as an effect of different dimensions of social determinants of health (quality of life index, primary health care index using principal component analysis to measure coverage of the program, physician ratio per population, nurse ratio per population, community health worker ratio per population; percentage of sewerage coverage; and the percentage insurance coverage of the health system), comparing the results in 2003 and 2007 (2) Study measured and compared the degree of socioeconomic-related inequality in the outcomes between 2003-2007 (3) Study measured the degree of contributions the primary health care contributes to the socioeconomic-related inequality (the dimensions of each social determinants of health in each outcome)
Nelson & Fritzell 2014<br><br>*Welfare states and population health: The role of minimum income benefits for mortality*	**Policy**	Generosity of minimum income benefits
	**Policy Area**	Social Protection and Welfare
	**Policy Target**	Upstream
	**Geographic Scope**	Cross-National Comparison (N=18)
	**Dataset(s)**	Repeated cross-sectional data; Multiple dataset; 1990-2009; Secondary data
	**Analysis**	Descriptive analysis: statistical summaries; Inferential analysis: fixed effects regression model
	**Outcome(s)**	<u>Long term</u>: age-standardised death rates, life expectancy at birth
	**Inequity Dimension**	Income + Sex

	**Summary**	Study measured associations between the generosity of minimum income benefits (low/intermediate/high), and age standardised death rates and life expectancy, comparing 18 OECD countries among men and women
Palència et al. 2014<br><br>*The influence of gender equality policies on gender inequalities in health in Europe*	**Policy**	Different types of family policy models that promote equal gender opportunities in labour and family protection
	**Policy Area**	Social Protection and Welfare
	**Policy Target**	Upstream
	**Geographic Scope**	Cross-National Comparison (N=26)
	**Dataset(s)**	Cross-sectional; Single dataset; 2010; Secondary data
	**Analysis**	Descriptive analysis: graphical analysis + statistical summaries; Inferential analysis: multivariate regression model + multilevel regression model
	**Outcome(s)**	Long-term: age-standardised self-perceived health
	**Inequity Dimension**	Age + Education + Occupation
	**Summary**	Study measured the association between the orientation of public gender equality policies and gender equalities in health by 1) comparing the prevalence ratios and age-standardised prevalence of poor self-perceived health among women and men, stratified by country and country typology of family policies (dual earner, traditional-central, traditional-southern, market oriented, contradictory) 2) comparing the prevalence ratio of poor self-perceived health according to gender, stratified by age, born in low-income country, education level, marriage status and employment status
Reeves et al. 2015<br><br>*Financing universal health coverage—effects of alternative tax structures on public health systems: cross-national modelling in 89 low-income and middle-income countries*	**Policy**	Different types of taxation revenue (progressive + regressive)
	**Policy Area**	Taxation
	**Policy Target**	Upstream
	**Geographic Scope**	Cross-National Comparison (N=89)
	**Dataset(s)**	Repeated cross-sectional; Multiple datasets; 1995-2011; Secondary data
	**Analysis**	Descriptive analysis: graphical analysis + statistical summaries; Inferential analysis: fixed effects regression model
	**Outcome(s)**	Intermediate: healthcare spending (public + private); access to health insurance<br>Long-term: maternal-child mortality rates
	**Inequity Dimension**	Income: low- and middle-income countries
	**Summary**	Study measured the associations between progressive and regressive revenue sources; and public and private health spending; and proportion of population with access to antenatal coverage, skilled births, and health coverage; and neonatal, post-neonatal, and under 5 mortality
Shahidi et al. 2016	**Policy**	Flexicurity policies (policies that encourage labour market entry and re-entry, promote employability and adaptability among workers, and provide inclusive social protection systems)

Do flexicurity policies protect workers from the adverse health consequences of temporary employment? A cross-national comparative analysis	**Policy Area**	Social Protection and Welfare
	**Policy Target**	Upstream
	**Geographic Scope**	Cross-National Comparison (N=23)
	**Dataset(s)**	Repeated cross-sectional; Single dataset (biennial); 2008-2012; Secondary data
	**Analysis**	Descriptive analysis: graphical analysis + statistical summaries; Inferential analysis: multilevel regression model
	**Outcome(s)**	Long-term: self-reported health and limiting longstanding illness
	**Inequity Dimension**	Occupation - precarious employment
	**Summary**	Studies compared the prevalence of poor self-reported health and longstanding limiting illnesses between permanent and temporary workers stratified by the flexibility and security of country-level labour market policies to assess whether flexicurity policies moderate the association between temporary employment and health
Shahidi, Siddiqi & Muntaner 2016<br><br>*Does social policy moderate the impact of unemployment on health? A multilevel analysis of 23 welfare states*	**Policy**	Social protection policies
	**Policy Area**	Social Protection and Welfare
	**Policy Target**	Upstream
	**Geographic Scope**	Cross-National Comparison (N=23)
	**Dataset(s)**	Cross-sectional; Single dataset; 2012; Secondary data
	**Analysis**	Descriptive analysis: statistical summaries; Inferential analysis: analysis of variance + multivariate regression model + multilevel regression model
	**Outcome(s)**	Intermediate: unemployment status<br>Long-term: self-rated health
	**Inequity Dimension**	Occupation - unemployed
	**Summary**	Study examined if the odds of reporting poor health among the unemployed are smaller in countries characterised by more generous levels of social protection for the unemployed and more favourable public attitudes towards the welfare state
**OBSERVATIONAL – CASE-CONTROL**		
Basu et al. 2016<br><br>*Health Behaviors, Mental Health, and Health Care Utilization Among Single Mothers*	**Policy**	1996 welfare reforms ("from welfare to work" programme)
	**Policy Area**	Social Protection and Welfare
	**Policy Target**	Upstream
	**Geographic Scope**	Localised (USA)
	**Dataset(s)**	Cohort; Single dataset (annual); 1993-2012; Secondary data

After Welfare Reforms in the 1990s	**Analysis**	Descriptive analysis: graphical analysis + statistical summaries; Inferential analysis: difference-in-difference-in-difference regression model
	**Outcome(s)**	Intermediate: tobacco smoking; binge alcohol consumption; medical access; affordability of medical access; routine Pap test and mammography<br>Long-term: mental health
	**Inequity Dimension**	Occupation - unemployed + Sex - single-mothers
	**Summary**	Study measured the association between welfare reforms and the probability of tobacco smoking; binge alcohol consumption; mental health; medical access; affordability of medical access; and routine Pap test and mammography screening between single mothers and the "control" group (married mothers, single nonmothers, and married nonmothers); between single mothers and a synthetic control group; and between employed and unemployed single mothers
Korenbrot, Kao & Crouch 2009<br><br>*Funding of tribal health programs linked to lower rates of hospitalization for conditions sensitive to ambulatory care*	**Policy**	Changes in Indian Health Service funding models of Tribally Operated Health Programs
	**Policy Area**	Health System Management/Health Insurance
	**Policy Target**	Upstream
	**Geographic Scope**	Localised - California, US
	**Dataset(s)**	Longitudinal; Multiple dataset; 1998-2002; Secondary data (linked hospital records)
	**Analysis**	Descriptive analysis: statistical summaries; Inferential analysis: generalised estimating equations regression model + multilevel regression model
	**Outcome(s)**	Long-term: Hospitalization rate for ambulatory-care sensitive conditions
	**Inequity Dimension**	Education + Income - poverty + Occupation - unemployed
	**Summary**	Study measured the association between higher funding of Tribally Operated Health Programs and reduced rate ratio of the hospitalisations for ambulatory care sensitive conditions, comparing among federal funding and medical price index, eligible population, socioeconomic status and resources, disparities in occupation, education, and household differences between indigenous and white population, Indian gaming revenue
**OBSERVATIONAL – LONGITUDINAL/REPEATED CROSS-SECTIONAL**		
Akin, Dow & Lance 2004<br><br>*Did the distribution of health insurance in China continue to grow less equitable in the nineties? Results from a longitudinal survey*	**Policy**	Health insurance reform (new insurance delivery mechanism)
	**Policy Area**	Health System Management/Health Insurance
	**Policy Target**	Upstream
	**Geographic Scope**	Localised - China
	**Dataset(s)**	Longitudinal; Single dataset; 1989-1997; Secondary data
	**Analysis**	Descriptive analysis: statistical summaries; Inferential analysis: multivariate regression model

	**Outcome(s)**	Intermediate: Health insurance coverage
	**Inequity Dimension**	Age - seniors + Education + Income - wealth + Occupation + Place of residence - rural/urban + Sex
	**Summary**	Study measured the association between health insurance reform and the likelihood of having insurance coverage at each time point (1989, 1991, 1993, 1997) and across all time, comparing location, urbanicity, sex, occupation and type of employer, age, education level and wealth.
Barr et al. 2015<br><br>*First, do no harm': are disability assessments associated with adverse trends in mental health? A longitudinal ecological study*	**Policy**	Work Capability Assessment on disability-related social security benefits
	**Policy Area**	Social Protection and Welfare
	**Policy Target**	Upstream
	**Geographic Scope**	Localised (England)
	**Dataset(s)**	Repeated cross-sectional; Multiple datasets (quarterly); 2004-2013; Secondary data
	**Analysis**	Descriptive analysis: graphical analysis + statistical summaries; Inferential analysis: fixed effects regression model
	**Outcome(s)**	Long term: suicides; anti-depressant prescriptions; self-reported mental health
	**Inequity Dimension**	Occupation - unemployed + Place of residence - disadvantaged area
	**Summary**	Study measured the association between proportion of population reassessed in each local authority area over time from 2010-2013 and the change in suicides rates and prevalence rates of self-reported mental health/antidepressant prescribing rates
Barr, Bambra & Whitehead 2014<br><br>*The impact of NHS resource allocation policy on health inequalities in England 2001-11: longitudinal ecological study*	**Policy**	Increasing National Health Service (NHS) funding to a greater extend in deprived areas
	**Policy Area**	Health System Management/Health Insurance
	**Policy Target**	Upstream
	**Geographic Scope**	Intra-National Comparison: England
	**Dataset(s)**	Repeated cross-sectional; Multiple dataset; 2001-2011; Secondary data
	**Analysis**	Descriptive analysis: statistical summaries; Inferential analysis: multivariate regression analysis
	**Outcome(s)**	Long term: mortality from causes amendable to healthcare
	**Inequity Dimension**	Place of residence - disadvantaged area
	**Summary**	Study measured the association between change in NHS allocation per head of population and mortality from causes amendable to healthcare, comparing the trends in equality between the most deprived and affluent area in relative and absolute terms among male and females
Blomqvist, Burström & Backhans 2014	**Policy**	Social insurance policy reform at times of economic recession (sickness insurance, unemployment insurance and tax reduction to encourage back to work)
	**Policy Area**	Social Protection and Welfare

Increasing health inequalities between women in and out of work-the impact of recession or policy change? A repeated cross-sectional study in Stockholm county, 2006 and 2010	**Policy Target**	Upstream
	**Geographic Scope**	Localised - Stockholm
	**Dataset(s)**	Repeated cross-sectional + longitudinal; Multiple dataset; Pre:2006 and post:2010; secondary data
	**Analysis**	Descriptive analysis: statistical summaries; Inferential analysis: multivariate regression analysis
	**Outcome(s)**	Long term: Mental distress, limiting longstanding illness
	**Inequity Dimension**	Occupation - unemployed + Sex
	**Summary**	Study measured the association between labour market position and health by comparing the odd ratios of women having mental distress and limiting longstanding illness between their labour market position (employment, unemployed, on sick leave, and on disability pension) before and after the social insurance reform
Boudreaux, Golberstein & McAlpine 2016<br><br>*The long-term impacts of Medicaid exposure in early childhood: Evidence from the program's origin*	**Policy**	Medicaid (public health insurance)
	**Policy Area**	Health System Management/Health Insurance
	**Policy Target**	Upstream
	**Geographic Scope**	Localised (USA)
	**Dataset(s)**	Longitudinal + Cohort; Multiple datasets; 1968-2009; Secondary data
	**Analysis**	Descriptive analysis: graphical analysis + statistical summaries; Inferential analysis: fixed effects regression model + difference-in-difference-in-difference regression model
	**Outcome(s)**	Intermediate: economic index (years of completed education, ratio of family income to the federal poverty line, and family wealth); health services utilization; medical debt<br>Long-term: composite chronic condition index (high blood pressure, diabetes, heart disease/heart attack, and obesity)
	**Inequity Dimension**	Age: youth + Income
	**Summary**	(1) Study measured the association between exposure (duration and timing) to Medicaid during early childhood on economic attainment and adult health stratified by income and predicted participation (2) Study measured the association between introduction of Medicaid and health services utilization for children; and family medical debt as an intermediate mechanism
Egan et al. 2016<br><br>*Proportionate universalism in practice? A quasi-experimental study (GoWell) of a UK neighbourhood renewal programme's impact on health inequalities*	**Policy**	City-wide investment allocation in neighbourhood renewal (housing improvement, social program, demolition and new build) according to area population needs
	**Policy Area**	Environment/Living Conditions
	**Policy Target**	Upstream
	**Geographic Scope**	Localised - Glasgow
	**Dataset(s)**	Cohort; Multliple dataset; Pre:2006, Post: 2011; Secondary data
	**Analysis**	Descriptive analysis: statistical summaries; Inferential analysis: difference-in-difference regression model

	**Outcome(s)**	Long term: Self-reported mental and physical health
	**Inequity Dimension**	Place of residence - amount of investment level
	**Summary**	(1) Study measured the association to which investment was allocated to need by comparing the investment amount per household to their income deprivation decile (2) Study measured the association between the mental and physical health and the level of investments by comparing the mean scores of mental and physical health with lower, medium and higher level of investments (3) Study measured the association of level of investment and change in the mean score of mental and physical health, comparing between the pre- and post-scores, stratified by 3 investment groups of lower, medium and higher level.
Stafford et al. 2008<br><br>*Tackling inequalities in health: Evaluating the New Deal for Communities initiative*	**Policy**	Neighbourhood Renewal program (focus on disadvantaged areas to reduce health inequalities)
	**Policy Area**	Environment/Living Conditions
	**Policy Target**	Mixed
	**Geographic Scope**	Intra-National Comparison: England
	**Dataset(s)**	Longitudinal; Single dataset; 2002, 2004; Primary data
	**Analysis**	Descriptive analysis: graphical analysis + statistical summaries; Inferential analysis: multilevel regression model
	**Outcome(s)**	Short term: fear of crime (person/property); satisfaction with local area<br>Intermediate: fruit and vegetable consumption; leisure-time physical activity; paid employment; annual personal income; participation in education/training; smoking status<br>Long term: self-rated general health; mental health; long-term limiting illness
	**Inequity Dimension**	Age + Education + Place of residence - disadvantaged area + Race/Ethnicity + Sex+ Socioeconomic status
	**Summary**	Study measured the association between the neighbourhood renewal program and the mean change in each outcomes, comparing those living in the intervention area and comparator areas, stratified by sex, age, education and ethnicity
Stafford et al. 2014<br><br>*Evaluating the health inequalities impact of area-based initiatives across the socioeconomic spectrum: a controlled intervention study of the New Deal for Communities, 2002–2008*	**Policy**	Neighbourhood Renewal program (focus on disadvantaged areas to reduce health inequalities)
	**Policy Area**	Environment/Living Conditions
	**Policy Target**	Mixed
	**Geographic Scope**	Intra-National Comparison: England
	**Dataset(s)**	Cohort + repeated cross-sectional; Multiple data (biannual + annual); 2002-2008; Secondary data
	**Analysis**	Descriptive analysis: graphical analysis + statistical summaries; Inferential analysis: multilevel regression model
	**Outcome(s)**	Intermediate: smoking status, employment status, housing tenure, education qualification<br>Long term: Mental health, self-rated health
	**Inequity Dimension**	Place of residence - disadvantaged area + Socioeconomic status

	**Summary**	Study compared the likelihood and the rate of change of the population smoking, having poor self-rated health, not in paid work, renting their accommodation, and with no qualification in areas that are equally poor but with and without the intervention, and in areas across the different socioeconomic spectrum (low/medium/high)
Stuckler, Basu & McKee 2011<br><br>*Health care capacity and allocations among South Africa's provinces: infrastructure-inequality traps after the end of apartheid*	**Policy**	Changes to allocations of health funds, and Growth, Employment and Redistribution strategy (focus on privatisation and fiscal austerity)
	**Policy Area**	Health System Management/Health Insurance
	**Policy Target**	Upstream
	**Geographic Scope**	Intra-National Comparison: Provinces in South Africa
	**Dataset(s)**	Repeated cross-sectional; Multiple data (annual); 1996-2007; Secondary data
	**Analysis**	Descriptive analysis: graphical analysis + statistical summaries; Inferential analysis: multivariate regression model
	**Outcome(s)**	Intermediate: Health care system (number of hospitals, doctors, nurses), total per capital public health spending, Treasury formula for economic activity<br>Long term: Burden of disease (antenatal HIV prevalence, infant mortality, crude death rates)
	**Inequity Dimension**	Place of residence
	**Summary**	(1) Study measured the trend of association between public health care allocations and measures of disease burden (antenatal HIV prevalence and infant mortality), income per capital and race over time (2) Study measured the association between public health allocations and burden of disease (antenatal HIV prevalence, infant mortality and crude death rate),
**INTERVENTIONAL – PRE-EXPERIMENTAL**		
Aghajanian et al. 2007<br><br>*Impact of rural health development programme in the Islamic Republic of Iran on rural-urban disparities in health indicators*	**Policy**	Community-based primary health care (PHC) system (started early 80s, expanded in 90s)
	**Policy Area**	Health System Management/Health Insurance
	**Policy Target**	Upstream
	**Geographic Scope**	Localised (Iran)
	**Dataset(s)**	Repeated cross-sectional; Multiple datasets; Pre: 1976 and Post: 2000; Secondary data
	**Analysis**	Descriptive analysis: statistical summaries
	**Outcome(s)**	Long-term: basic health indicators (mortality rates, life expectancy), outcomes varied from pre to post intervention
	**Inequity Dimension**	Place of residence - rural/urban + Sex
	**Summary**	Study measured changes in health indicators before and after a rural PHC programme stratified by men and women and urban and rural
Edwards et al. 2008	**Policy**	Smoke-free Environments<br>Amendment Act (2003)

After the smoke has cleared: evaluation of the impact of a new national smoke-free law in New Zealand	**Policy Area**	Environment/Living Conditions
	**Policy Target**	Upstream
	**Geographic Scope**	Localised (New Zealand)
	**Dataset(s)**	Repeated cross-sectional; Multiple datasets; 1996-2006; Secondary data
	**Analysis**	Descriptive analysis: graphical analysis + statistical summaries
	**Outcome(s)**	Short-term: compliance with legislation; changes in knowledge, attitudes and beliefs<br>Intermediate: reduced smoking and exposure to second-hand smoke, changes in opportunities to smoke, desire to start or stop; reduced smoking prevalence and consumption<br>Long-term: reduced health effects
	**Inequity Dimension**	Income + Race/Ethnicity - Indigenous
	**Summary**	Study compared support for smoking bans in bars and restaurants, compliance with smoke-free environments, second hand smoke exposure, hospitalisation rates for acute asthma, acute stroke, unstable angina, and exacerbations of chronic obstructive pulmonary disease before and after policy implementation stratified by sex and indigenous status (depending on available data)
Ganaba et al. 2016<br><br>*The obstetric care subsidy policy in Burkina Faso: what are the effects after five years of implementation? Findings of a complex evaluation*	**Policy**	National subsidy to reduce user fees for maternity services (2006)
	**Policy Area**	Health System Management/Health Insurance
	**Policy Target**	Upstream
	**Geographic Scope**	Localised (Burkina Faso)
	**Dataset(s)**	Repeated cross-sectional; Multiple datasets; 1988-2012; Primary and Secondary data
	**Analysis**	Descriptive analysis: graphical analysis + statistical summaries; Inferential analysis: multivariate regression model
	**Outcome(s)**	Short-term: awareness and user satisfaction<br>Intermediate: utilisation of services; quality of care; household costs; effects on staff and facilities
	**Inequity Dimension**	Income + Place of residence - rural/urban
	**Summary**	Study compared awareness and user satisfaction, utilisation of services, quality of care, household costs and effects on staff and facilities before and after policy implementation stratified by both urban and rural and wealth quintile
Jackson et al. 2011<br><br>*Reduced acute hospitalisation with the*	**Policy**	Healthy housing programme
	**Policy Area**	Environment/Living Conditions
	**Policy Target**	Upstream
	**Geographic Scope**	Localised (South Auckland, New Zealand)

healthy housing programme	**Dataset(s)**	Longitudinal; Single dataset; Pre, During, and Post-Intervention periods, individually determined by exposure from 1999-2009; Secondary data (hospital records)
	**Analysis**	Descriptive analysis: statistical summaries; Inferential analysis: survival analysis regression model
	**Outcome(s)**	Long-term: acute medical-surgical hospital admissions potentially related to housing; avoidable hospitalisations; mortality
	**Inequity Dimension**	Place of residence - disadvantaged area + Race/Ethnicity - Indigenous
	**Summary**	Study measured the association between healthy housing programme intervention and the hazard of hospitalisation, comparing the intervention group pre-, during-, and post-intervention stratified by age group
Lankarani et al. 2012<br><br>*Reducing Social Disparity in Liver Transplantation Utilization through Governmental Financial Support*	**Policy**	Government coverage of 100% of the costs related to liver transplantation surgery
	**Policy Area**	Health System Management/Health Insurance
	**Policy Target**	Upstream
	**Geographic Scope**	Localised (Iran)
	**Dataset(s)**	Repeated cross-sectional; Single dataset; Pre: 2003-2005 and Post 2005-2007; Primary data
	**Analysis**	Descriptive analysis: graphical analysis + statistical summaries; Inferential analysis: mean comparison + chi-square
	**Outcome(s)**	Long-term: liver transplant
	**Inequity Dimension**	Place of residence - disadvantaged area + Socioeconomic status
	**Summary**	Study compared the socioeconomic characteristics of patients (house and car ownership as proxies for income, level of education, employment status, and place of residence) treated by liver transplantation before and after the policy intervention
Liu et al. 2002<br><br>*Equity in health care access to: assessing the urban health insurance reform in China*	**Policy**	Urban health insurance reform
	**Policy Area**	Health System Management/Health Insurance
	**Policy Target**	Upstream
	**Geographic Scope**	Localised (Zhenjiang, China)
	**Dataset(s)**	Repeated cross-sectional; Single dataset (annual); Pre: 1993-1994 and Post:1995-1996; Secondary data
	**Analysis**	Descriptive analysis: statistical summaries; Inferential analysis: difference-in-difference regression model
	**Outcome(s)**	Intermediate: health care utilization
	**Inequity Dimension**	Health - poor health + Socioeconomic status
	**Summary**	Study compared the changes in the likelihood of obtaining various health care services across sub-population groups with different socioeconomic status and health conditions before and after the policy intervention
Liu, Wong & Liu 2016	**Policy**	Health insurance reforms (2009)

Outcome-based health equity across different social health insurance schemes for the elderly in China	**Policy Area**	Health System Management/Health Insurance
	**Policy Target**	Upstream
	**Geographic Scope**	Localised (China)
	**Dataset(s)**	Repeated cross-sectional; Single dataset; Pre: 2006 and Post: 2010; Secondary data
	**Analysis**	Descriptive analysis: statistical summaries; Inferential analysis: analysis of variance + multivariate regression model
	**Outcome(s)**	Intermediate: health care expenditure<br>Long-term: health status (self-reported health status, physical function and psychological wellbeing)
	**Inequity Dimension**	Socioeconomic status
	**Summary**	Study compared the health care expenditure and health status of the elderly before and after health care reform stratified by insurance coverage plan, age, sex, education, and income
Moysés et al. 2006<br><br>*Intra-urban differentials in child dental trauma in relation to Healthy Cities policies in Curitiba, Brazil*	**Policy**	World Health Organisation Healthy Cities Project
	**Policy Area**	Environment/Living Conditions
	**Policy Target**	Mixed
	**Geographic Scope**	Localised (Curitiba, Brazil)
	**Dataset(s)**	Repeated cross-sectional; Multiple datasets;1990-2000; Primary data
	**Analysis**	Descriptive analysis: graphical analysis + statistical summaries + principal components analysis; Inferential analysis: chi-square analysis + hierarchical regression model + meta-regression model
	**Outcome(s)**	Long-term: prevalence of dental trauma in 12-year-old schoolchildren
	**Inequity Dimension**	Place of residence - disadvantaged area
	**Summary**	Study measured the differences in prevalence of dental traumas between areas that were demonstrated to be "better-off" (top 50% of areas) after the Healthy Cities Project implementation relative to the areas that were demonstrated to be "worse-off" (bottom 50% of areas)
Vellakkal et al. 2016<br><br>*Has India's national rural health mission reduced inequities in maternal health services? A pre-post repeated cross-sectional study*	**Policy**	India National Rural Health Mission (cash-transfer programme for women in low SES to give birth in a health facility)
	**Policy Area**	Social Protection and Welfare
	**Policy Target**	Upstream
	**Geographic Scope**	Intra-National Comparison: India
	**Dataset(s)**	Repeated cross-sectional; Multiple dataset; Pre:1995-2004, Post: 2007-2012; Secondary data
	**Analysis**	Descriptive analysis: graphical analysis + statistical summaries; Inferential analysis: difference-in-difference regression model
	**Outcome(s)**	Intermediate: update of institutional delivery, ante-natal care

	**Inequity Dimension**	Education + Income - wealth + Place of residence - rural/urban + Sex
	**Summary**	Study compared the wealth-related and education-related relative index of inequality in the uptake of institutional delivery and antenatal care pre- and post-policy intervention between the high-focus empowered action group states and north eastern Indian states
Xu et al. 2007<br><br>*Urban health insurance reform and coverage in China using data from National Health Services Surveys in 1998 and 2003*	**Policy**	Urban health insurance reform
	**Policy Area**	Health System Management/Health Insurance
	**Policy Target**	Upstream
	**Geographic Scope**	Localised (China)
	**Dataset(s)**	Repeated cross-sectional; Single dataset; 1998 and 2003; Secondary data
	**Analysis**	Descriptive analysis: statistical summaries
	**Outcome(s)**	Intermediate: health insurance coverage for the urban population
	**Inequity Dimension**	Age - youth and seniors + Income + Occupation - unemployed + Sex
	**Summary**	Study compared the health insurance coverage for the urban population in the years before and after the policy reforms stratified by age, employment status, income level, and gender
**INTERVENTIONAL – NATURAL EXPERIMENT**		
Dow & Schmeer 2003<br><br>*Health insurance and child mortality in Costa Rica*	**Policy**	Health insurance expansion (focus on children)
	**Policy Area**	Health System Management/Health Insurance
	**Policy Target**	Upstream
	**Geographic Scope**	Localised - Costa Rica
	**Dataset(s)**	Repeated cross-sectional; Multiple dataset; Pre: 1973, post: 1984; Secondary data
	**Analysis**	Descriptive analysis: graphical analysis + statistical summaries; Inferential analysis: fixed effects regression model
	**Outcome(s)**	Long term: Infant and child mortality
	**Inequity Dimension**	Education + Health - availability of health services + Income - wealth + Sex - single mother
	**Summary**	Study measured the associations between child insurance coverage and infant mortality; and child mortality, comparing among mother education level, married status, migration status, household characteristic, household wealth, and county healthcare infrastructure
Edwards et al. 2013	**Policy**	Free bus travel for youth (age 12–17)
	**Policy Area**	Social Protection and Welfare
	**Policy Target**	Upstream

Health impacts of free bus travel for young people: evaluation of a natural experiment in London	**Geographic Scope**	Localised (London, UK)
	**Dataset(s)**	Repeated cross-sectional; Multiple datasets; Pre:2001-2004 and Post:2006-2009; Secondary data
	**Analysis**	Descriptive analysis: graphical analysis + statistical summaries; Inferential analysis: change-on-change regression model
	**Outcome(s)**	Intermediate: frequency of bus travel, active travel, car travel; transport poverty; transport injury; assault; car dependence; access to education, training, independent travel; effects on older citizens<br>Long-term: physical/mental health
	**Inequity Dimension**	Age - youth + Income - deprivation + Race/Ethnicity
	**Summary**	(1) Study compared bus, car, and active transport use; road injuries; and assaults pre- and post-policy intervention between youth and adults (2) Study compared distances travelled by youth according to journey purpose by decile of deprivation pre- and post-policy intervention (3) Study compared distances walked and cycled; road injuries; and assaults by area of London, deprivation, household income, ethnicity, and intervention uptake pre- and post-policy intervention between youth and adults
Goodman et al. 2013<br><br>*Effectiveness and equity impacts of town-wide cycling initiatives in England: a longitudinal, controlled natural experimental study*	**Policy**	Town- and city-wide interventions to increase cycling
	**Policy Area**	Environment/Living Conditions
	**Policy Target**	Mixed
	**Geographic Scope**	Intra-National Comparison: England
	**Dataset(s)**	Repeated cross-sectional; Single dataset (decennial); Pre:2001, Post: 2011; Secondary data
	**Analysis**	Descriptive analysis: graphical analysis + statistical summaries; Inferential analysis: difference-in-difference regression model + meta-regression model
	**Outcome(s)**	Intermediate: Frequency of cycling, walking, driving, and use of public transport to work
	**Inequity Dimension**	Place of residence - disadvantaged area
	**Summary**	1) Study measured the association between the cycling initiative interventions and change in prevalence of cycling; walking; driving; and taking public transport to work, comparing the pre- and post-intervention absolute and relative changes in the intervention group with matched comparison groups; unfunded comparison groups; non-London national comparison groups 2) Study measured the association between small-area deprivation and prevalence of cycling to work, comparing the percentage-point change and ratio of change in the intervention group with matched comparison groups; unfunded comparison groups; non-London national comparison groups stratified by deprivation indices
Kearns & Mason 2015	**Policy**	Housing investment, transformational area regeneration programme, and relocation due to demolition and rebuilding
	**Policy Area**	Environment/Living Conditions
	**Policy Target**	Upstream

Regeneration, relocation and health behaviours in deprived communities	**Geographic Scope**	Intra-National Comparison: Glasgow, UK
	**Dataset(s)**	Cross-sectional; Single data; 2011; Secondary data
	**Analysis**	Descriptive analysis: statistical summaries; Inferential analysis: multivariate regression model
	**Outcome(s)**	Intermediate: smoking behaviour and habits, drinking habits, dietary consumption, physical activity
	**Inequity Dimension**	Place of residence - disadvantaged area
	**Summary**	Study assigned samples into 3 groups based on their current location, residential circumstances and receiving regeneration in their living areas (since 2008): those living in non-regeneration area and not receiving any improvements (nonmovers); those who have remained living in the same dwellings but received regeneration (remainers); and those who have moved out of the regeneration areas into a social-rented dwellings (outmovers). Study compared the odd ratios of each outcomes in the 3 comparison groups: between remainers and outmovers relative to nonmovers; and outmovers relative to remainers, each comparison stratified by dwelling quality, neighbourhood quality, sex, education level, occupation, long-standing illness, and household types
Kelaher, Warr & Tacticos 2010<br><br>*Evaluating health impacts: Results from the neighbourhood renewal strategy in Victoria, Australia*	**Policy**	Neighbourhood Renewal (focus on disadvantaged neighbourhoods to reduce inequalities across a range of social, educational and health outcomes)
	**Policy Area**	Environment/Living Conditions
	**Policy Target**	Mixed
	**Geographic Scope**	Localised (Victoria, Australia)
	**Dataset(s)**	Repeated cross-sectional; Single dataset (pre and post measurement); No date; Primary data
	**Analysis**	Descriptive analysis: graphical analysis + statistical summaries; Inferential analysis: multivariate regression model
	**Outcome(s)**	Long-term: self-reported health and life satisfaction
	**Inequity Dimension**	Place of residence - disadvantaged area
	**Summary**	Study measured the association between involvement in the intervention and health/life satisfaction outcomes over time, comparing people who were involved in the intervention and people who were not involved in the intervention to the control group in the same local government area
Reeves et al. 2014<br><br>*Do employment protection policies reduce the relative disadvantage in the labour market experienced by unhealthy people? A natural experiment*	**Policy**	Employment protection policies
	**Policy Area**	Social Protection and Welfare
	**Policy Target**	Upstream
	**Geographic Scope**	Cross-National Comparison (N=26)
	**Dataset(s)**	Longitudinal, Single dataset (annual); Pre: 2006-2008 and Post: 2008-2010; Secondary data
	**Analysis**	Descriptive analysis: graphical analysis + statistical summaries; Inferential analysis: multilevel regression model
	**Outcome(s)**	Intermediate: unemployment status

created by the Great Recession in Europe	**Inequity Dimension**	Health - poor health
	**Summary**	Study compared the odds of job loss prior to a recession and after a mild or severe recession in individuals without and without health limitations, and with and without a chronic illness and the protective effect of employment protection polices stratified by sex
Reeves et al. 2016<br><br>*Introduction of a National Minimum Wage Reduced Depressive Symptoms in Low-Wage Workers: A Quasi-Natural Experiment in the UK*	**Policy**	Minimum wage legislation
	**Policy Area**	Social Protection and Welfare
	**Policy Target**	Upstream
	**Geographic Scope**	Localised (UK)
	**Dataset(s)**	Longitudinal; Single dataset (annual); Pre: 1994-1998 and Post:1999-2001; Secondary data
	**Analysis**	Descriptive analysis: graphical analysis + statistical summaries; Inferential analysis: difference-in-difference regression model
	**Outcome(s)**	Long-term: self-reported mental health, physical health (elevated blood pressure, cigarettes smoked)
	**Inequity Dimension**	Occupation - low-payed employment
	**Summary**	Study compared the mental and physical health changes in people who received a wage increase after introduction of the policy intervention to those who did not receive the policy intervention
**INTERVENTIONAL – QUASI EXPERIMENTAL**		
Kloek et al. 2006<br><br>*Impact evaluation of a Dutch community intervention to improve health-related behaviour in deprived neighbourhoods*	**Policy**	Healthy neighbourhood programme (Focus on health promotion and community empowerment)
	**Policy Area**	Environment/Living Conditions
	**Policy Target**	Midstream
	**Geographic Scope**	Localised (Eindhovan, Netherlands)
	**Dataset(s)**	Longitudinal; Single dataset; pre:2002, post:2002; primary data
	**Analysis**	Descriptive analysis: graphical analysis + statistical summaries; Inferential analysis: analysis of variance + multivariate regression model + generalised estimating equations regression model
	**Outcome(s)**	*Short term*: Fruit and vegetable knowledge; attitude, self-efficacy expectations, awareness and changes in fruit, vegetable and physical activity<br>Intermediate: daily fruit and vegetable intake; level of physical activity; tobacco and alcohol consumption
	**Inequity Dimension**	Place of residence - disadvantaged area
	**Summary**	Study measured the associations between the intervention and the outcomes of health-related behaviour, comparing between 3 intervention neighbourhoods and 3 matched comparison neighbourhoods.
**INTERVENTIONAL – EXPERIMENTAL**		

Van de gaer, Vandenbossche & Figueroa 2014<br><br>*Children's Health Opportunities and Project Evaluation: Mexico's Oportunidades Program*	**Policy**	Conditional cash transfer program for compliance with preventive health care
	**Policy Area**	Social Protection and Welfare
	**Policy Target**	Upstream
	**Geographic Scope**	Localised (Mexico)
	**Dataset(s)**	Longitudinal; Single dataset; Pre:1997 Post:2003; Secondary data
	**Analysis**	Descriptive analysis: graphical analysis + statistical summaries; Decision analysis: stochastic dominance
	**Outcome(s)**	Long-term: malnutrition (anaemia and stunting); obesity (BMI); number of days parents reported that the child was sick during the previous four-week period
	**Inequity Dimension**	Age - youth + Education + Race/Ethnicity - Indigenous
	**Summary**	Study measured whether the policy had a substantially favourable effect on the health opportunities of the most disadvantaged children, that is, those with parents of indigenous origin and without a parent who completed primary education
**MODELLING AND SIMULATION**		
Basu et al. 2014<br><br>*Averting Obesity and Type 2 Diabetes in India through Sugar-Sweetened Beverage Taxation: An Economic-Epidemiologic Modeling Study*	**Policy**	Sugar-sweetened beverage tax (20%)
	**Policy Area**	Taxation
	**Policy Target**	Upstream
	**Geographic Scope**	Localised (India)
	**Dataset(s)**	Repeated cross-sectional; Multiple datasets; 2007-2010; Secondary data
	**Analysis**	Descriptive analysis: graphical analysis + statistical summaries; Modelling analysis: microsimulation modelling
	**Outcome(s)**	Long-term: overweight, obesity, and type 2 diabetes rates
	**Inequity Dimension**	Age + Income + Place of residence - rural/urban + Sex
	**Summary**	Study modelled the projected impact of a 20% sugar-sweetened beverage tax on increases in overweight, obesity, and type 2 diabetes cases stratified by age, sex, income, and urban/rural residence
Blakely et al. 2014<br><br>*Cost-effectiveness and equity impacts of three HPV vaccination programmes for school-aged girls in New Zealand*	**Policy**	HPV vaccination programme
	**Policy Area**	Health System Management/Health Insurance
	**Policy Target**	Upstream
	**Geographic Scope**	Localised (New Zealand)
	**Dataset(s)**	Repeated cross-sectional; Multiple datasets; 1994-2011; Secondary data
	**Analysis**	Descriptive analysis: graphical analysis + statistical summaries; Modelling analysis: Markov macrosimulation modelling

	**Outcome(s)**	Intermediate: health system costs and cost-effectiveness<br>Long-term: health gains in QALY
	**Inequity Dimension**	Income - deprivation + Race/Ethnicity - Indigenous + Sex
	**Summary**	Study modelled the health gains, net-cost, and cost-effectiveness of three models of HPV national vaccination programmes in regards to HPV-related cancers stratified by sex, ethnicity, and area-based socioeconomic deprivation
Blakely et al. 2015<br><br>*Health, Health Inequality, and Cost Impacts of Annual Increases in Tobacco Tax: Multistate Life Table Modelling in New Zealand*	**Policy**	Tobacco tax<br>increases (10% annually)
	**Policy Area**	Taxation
	**Policy Target**	Upstream
	**Geographic Scope**	Localised (New Zealand)
	**Dataset(s)**	Repeated cross-sectional; Multiple datasets; 2006-2013; Secondary data
	**Analysis**	Descriptive analysis: graphical analysis + statistical summaries; Modelling analysis: multistate life table modelling
	**Outcome(s)**	Intermediate: health system costs<br>Long-term: health gains in QALY
	**Inequity Dimension**	Race/Ethnicity - Indigenous
	**Summary**	Study modelled the health gains and the health system costs of a 10% annual tobacco tax increase (relative to business as usual) in regards to tobacco-related diseases stratified by sex and ethnicity
Pearson et al. 2016<br><br>*Tobacco retail outlet restrictions: health and cost impacts from multistate life-table modelling in a national population*	**Policy**	4 interventions to restrict tobacco retail outlet (reduce total outlets by 95%, permit sales at 50% liquor outlets only, eliminate sales within 1km from schools, eliminate sales from 2km from schools)
	**Policy Area**	Environment/Living Conditions
	**Policy Target**	Upstream
	**Geographic Scope**	Localised (New Zealand)
	**Dataset(s)**	Cohort; Multiple data; 2006-2010; Secondary data
	**Analysis**	Descriptive analysis: statistical summaries; Modelling analysis: multistate life table modelling
	**Outcome(s)**	Long term: quality-adjusted life years gained; net health system cost-savings
	**Inequity Dimension**	Age + Race/Ethnicity - Indigenous + Sex
	**Summary**	(1) Study modelled the estimated changes in incidence, prevalence and case-death rates of 16 tobacco-related diseases from the 4 interventions (2) Study modelled the years of life saved, adjusting for disability from the change in disease rates (3) Study modelled the cost of cigarettes, factoring in travel cost by estimating change in distance and time travelling to reduced and location of retail outlets (3) Study measured change in disease rate factoring in price

		elasticities from changes in smoking behaviour (4) Study modelled QALY and cost-savings, comparing between business-as-usual- model and the 4 interventions, stratified by different population groups
**META-ANALYSIS**		
Ogilvie et al. 2008<br><br>*The harvest plot: a method for synthesising evidence about the differential effects of interventions.*	**Policy**	Population-level tobacco control interventions
	**Policy Area**	Environment/Living Conditions
	**Policy Target**	Mixed
	**Geographic Scope**	Not specified
	**Dataset(s)**	Studies which assessed the effects of any type of population-level tobacco control intervention and had reported effects stratified by at least one demographic or socio-economic characteristic irrespective of study design, methodological quality or outcomes measured (N=85)
	**Analysis**	Descriptive analysis: graphical analysis + statistical summaries; Meta-analysis: Harvest plot
	**Outcome(s)**	Intermediate: change in awareness, change in attitude, change in perceived availability<br>Long-term: change in self-reported smoking status
	**Inequity Dimension**	Age + Education + Income + Occupation + Race/Ethnicity + Sex
	**Summary**	Study systematically reviewed the results of studies on the effects of population-level tobacco control interventions on changes in awareness, attitude, perceived availability and self-reported smoking status stratified by income, occupation, education, gender, ethnicity, and age
